# Ghost shrimps (Decapoda: Axiidea: Callianassidae) as producers of an Upper Miocene trace fossil association from sublittoral deposits of Lake Pannon (Vienna Basin, Slovakia)

**DOI:** 10.1016/j.palaeo.2015.02.012

**Published:** 2015-05-01

**Authors:** Matúš Hyžný, Vladimír Šimo, Dušan Starek

**Affiliations:** a Department of Geology and Paleontology, Natural History Museum Vienna, Burgring 7, A-1010 Vienna, Austria; b Department of Geology and Palaeontology, Faculty of Natural Sciences, Comenius University, Mlynská dolina G1, SK-842 15 Bratislava, Slovakia; c Geological Institute, Slovak Academy of Sciences, Dúbravská cesta 9, SK-840 05 Bratislava, Slovakia

**Keywords:** *Egbellichnus jordidegiberti* igen et isp. nov, the Vienna Basin, Lake Pannon, Upper Miocene, callianassid ghost shrimp

## Abstract

Numerous trace fossils are described from the Late Miocene sediments of the Bzenec Formation exposed at the Gbely section (the Vienna Basin, Slovakia). During deposition of the sediments the area was part of the large, long-lived brackish to freshwater Lake Pannon. Most of the trace fossils are attributed herein to *Egbellichnus jordidegiberti* igen et ispec. nov. and are interpreted as burrows produced by decapod crustaceans, specifically by a ghost shrimp of the family Callianassidae. This interpretation is based on two independent lines of evidence: environmental requirements of large bioturbators and the burrow morphology itself. The new ichnotaxon is distinguished from other related ichnotaxa by a combination of typically inclined (roughly at an angle of 45°) cylindrical burrows, absence of lining, and tunnels making loops or bends at approximately right angles. The burrow systems at Gbely document the survival of ghost shrimp long after the closure of all seaways and the origin of Lake Pannon. As today, no ghost shrimp are known from long-lived brackish lakes. *Egbellichnus* from Gbely is the only, although indirect, record of ghost shrimp from a brackish lake environment reported so far.

## Highlights

• A new ichnotaxon *Egbellichnus jordidegiberti* igen et isp. nov is erected.
• A tracemaker is identified as a ghost shrimp (Callianassidae).
• *Egbellichnus* is the only record of ghost shrimps from the brackish lake environment reported so far.

## 1 Introduction

Decapod crustaceans are important elements of not only marine, but also of brackish and freshwater environments (Dworschak, 2000a; Felder, 2001; Crandall and Buhay, 2008; Yeo et al., 2008). Brackish water has a salinity range between 0.5–30 ‰; thus, it is not considered a precisely defined condition and covers a broad range of salinity regimes. For many brackish surface waters, salinity can vary considerably over space and/or time. Organisms inhabiting such an environment must tolerate these fluctuations. Together with molluscs and polychaetes (Wolff, 1973; Grassle and Grassle, 1974; Maggiore and Keppel, 2007), decapod crustaceans are among the most successful macroscopic invaders of brackish-water environments. Three major decapod groups made this transition: mud shrimps (Gebiidea: Upogebiidae), ghost shrimps (Axiidea: Callianassidae) and true crabs (Brachyura). Ghost shrimps nowadays constitute important components of the normal marine near-shore macro-invertebrate assemblages and also of estuarine environments with high salinity fluctuations (Dworschak, 2000a). True freshwater brachyurous crabs constitute nearly 20 % of all known extant crab species in 14 families (Yeo et al., 2008); some of them are able to live inland (Taylor and Greenaway, 1979; Waltham et al., 2014; Williner et al., 2014).

Many decapod taxa inhabiting brackish water environment produce burrows. Consequently, these can be identified in the sedimentological record as trace fossils with characteristic features. Numerous trace fossils found in the Late Miocene sediments of the Gbely section (the Vienna Basin, Slovakia) are interpreted herein as burrows produced by decapod crustaceans, specifically by ghost shrimp of the Callianassidae. The first report on burrows made by “thalassinideans” from the Gbely section can be found in Starek et al. (2010). The present contribution builds further on the sedimentological, palaeontological and geochemical results of Starek et al. (2010).

The objectives of this paper are 1) the report of the studied ichnoassemblage of the Gbely section; 2) description of a new ichnogenus and ichnospecies; 3) the interpretation of its tracemaker based on evidence from palaeoenvironmental reconstruction and burrow morphology; and 4) providing arguments for the presence of ghost shrimp in the long-lived brackish Lake Pannon.

## 2 Study area and methods

The Paratethys was an epicontinental sea that developed in the Early Oligocene as a consequence of Africa’s northward movement and resulting European plate subduction. It was intermittently connected to the Mediterranean and the Indo-Pacific (Rögl, 1998; Harzhauser and Piller, 2007; Harzhauser et al., 2007). The area from present-day Austria to Poland, Ukraine and Romania is called the Central Paratethys.

The Pannonian Basin formed in the late Early Miocene and in the Middle Miocene as a result of extension and rifting governed by thrusting in the surrounding Carpathian orogene (Horváth et al., 2006). At the end of the Middle Miocene a significant sea-level regression resulted in isolation of the intra-Carpathian waters from the rest of the Paratethys, forming the large, long-lived brackish to freshwater Lake Pannon (Magyar et al., 1999; Harzhauser and Mandic, 2008). It separated from the Central Paratethys at about 11.6 Ma (Magyar et al., 1999; Vasiliev, 2006; Harzhauser and Piller, 2007). Since that time, shallow brackish-marine conditions were typical of the Vienna Basin (Kováč et al., 1998, 2004, 2005; Magyar et al., 1999; Starek et al., 2010). The lake basin was filled by progradation from the NE and NW (Magyar et al., 2007). By the Early Pliocene, Lake Pannon became a shallow freshwater lake and gradually it was completely filled with sediment (Magyar et al., 1999).

The depositional systems of Lake Pannon are heterogeneous, represented by alluvial and fluvial facies, ephemeral lake, swamp, and subaquatic delta plain deposits passing continually to offshore pelitic facies (Harzhauser and Tempfer, 2004; Kováč et al., 2005).

### 2.1 Previous ichnological research

Trace fossils of the deposits of the Pannon Lake were reported by several authors. Szónoky (1978) reported unidentified burrows from both shallow and relatively deep sublittoral environments of the Lower Pannonian Formation (*sensu* Jámbor, 1980) characterized by fine-grained, gray, homogeneous, calcareous clays and marls. According to Jámbor (1980, 1987), the most abundant trace fossils in this formation are a Y-shaped form classified as “*Thalassinoides minimus*” and “*Pectinaria* burrows”; other common trace fossils are *Spirisiphonella pannonica* and *Arenicolites* isp. Trace fossils studied by Jámbor (1980, 1987), however, come from the boreholes and their relation to the ichnofacies as well as identification is dubious and need to be revised.

From the Szák Formation (the uppermost part of the Lower Pannonian Formation *sensu* Jámbor, 1980), Cziczer et al. (2009) reported *Diplocraterion* and “Y-shaped trace fossils”.

From the basal part of the Kálla Sand (Upper Pannonian Formation *sensu* Jámbor, 1980), *Arenicolites*, *Skolithos*, *Polykladichnus*, funnel-shaped trace fossils and burrows of vertebrates were reported by Babinszski et al. (2003). Funnel-shaped trace fossils were found with their producers *in situ* and were determined to be escape structures of the bivalve *Dreissenomya* (Magyar et al., 2006). This ichnoassemblage reflects an oxygenated environment with unstable substrate at depths at the fair-weather wave-base influenced by storms.

From the sediments of the Bzenec Formation exposed at Gbely section, Starek et al. (2010) reported several ichnofossils treated in the open nomenclature, namely “Thalassinidean (types 1 and 2) burrows” and “*Conichnus*-like conical ichnofossils”. “Thalassinidean burrows” have recently been further studied and extensively sampled and provide a basis for description of a new ichnotaxon erected herein.

### 2.2 Sedimentology and lithofacies of the Gbely section

**Fig. 1:**
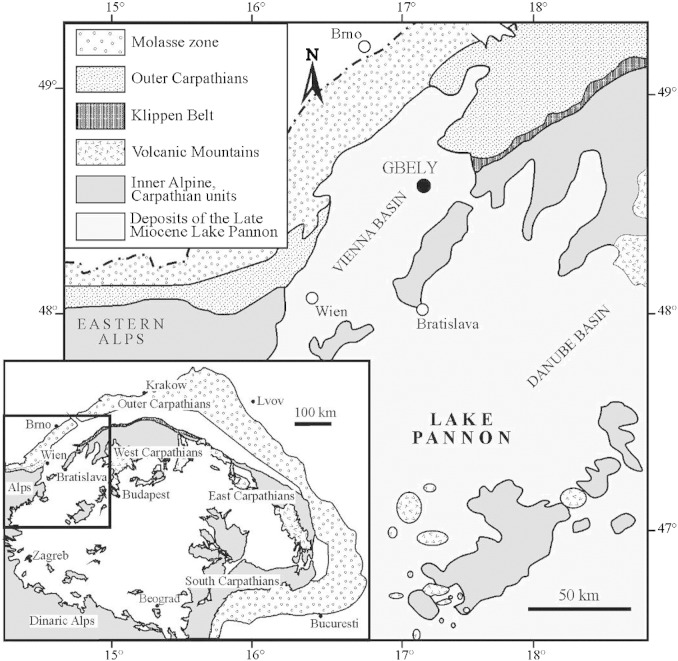
Study area.

The studied sedimentary sequence of fine-grained deposits is situated at the Gbely locality in the Slovak part of the Vienna Basin. The outcrop is situated in an abandoned brick-yard situated approximately 1.2 km SSE from the centre of the town of Gbely (Fig. 1; coordinates 48° 42' 26.33" N; 17° 07' 12.74" E). The lithofacies exposed in the brickyard is part of the lacustrine-deltaic succession belonging to the Záhorie Member of the Bzenec Formation deposited during the Late Miocene (Vass, 2002). The ostracod associations as well as autochthonous preservation of *Congeria subglobosa* shells at the studied outcrop (Starek et al., 2010) permitted assignment of the succession to the regional Pannonian zone E *sensu* Papp (1951). This zone is supported by the ostracods *Cyprideis obesa*, *C. heterostigma*, *Hemicytheria folliculosa*, and *H. reniformis* (Jiříček, 1985). The outcrop is biostratigraphically coeval with clay deposits exposed in Hennersdorf, Austria (Harzhauser and Mandic, 2004) where magnetostratigraphic measurements indicate interval C5n (11.04–9.78 Ma) (Magyar et al., 2007).

**Fig. 2:**
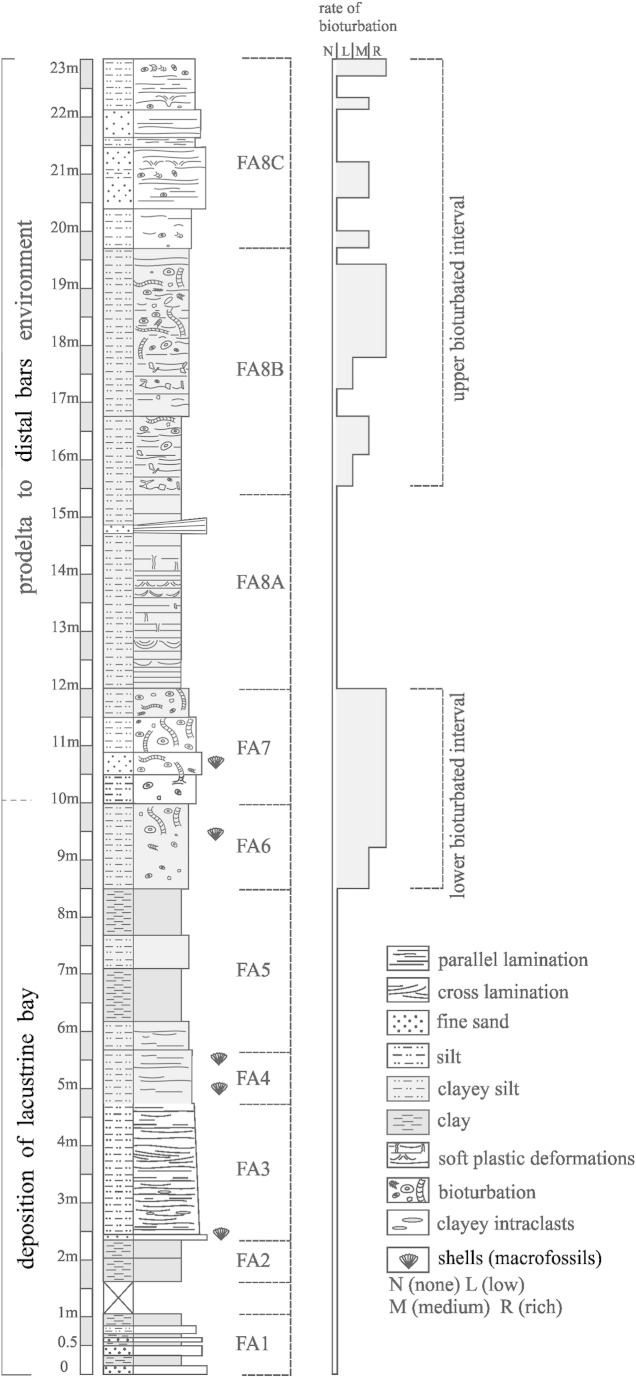
The studied section at Gbely (modified after Starek et al., 2010). Lower bioturbated interval = BI 1; upper bioturbated interval = BI 2. For details see text.

The studied sequence can be divided into four intervals (Fig. 2). Two of them are highly bioturbated (the lower and upper bioturbated horizons). Between them there is a sequence with soft-sediment deformation structures. These intervals correspond to diverse facies associations (FA) *sensu* Starek et al. (2010).

**Fig. 3:**
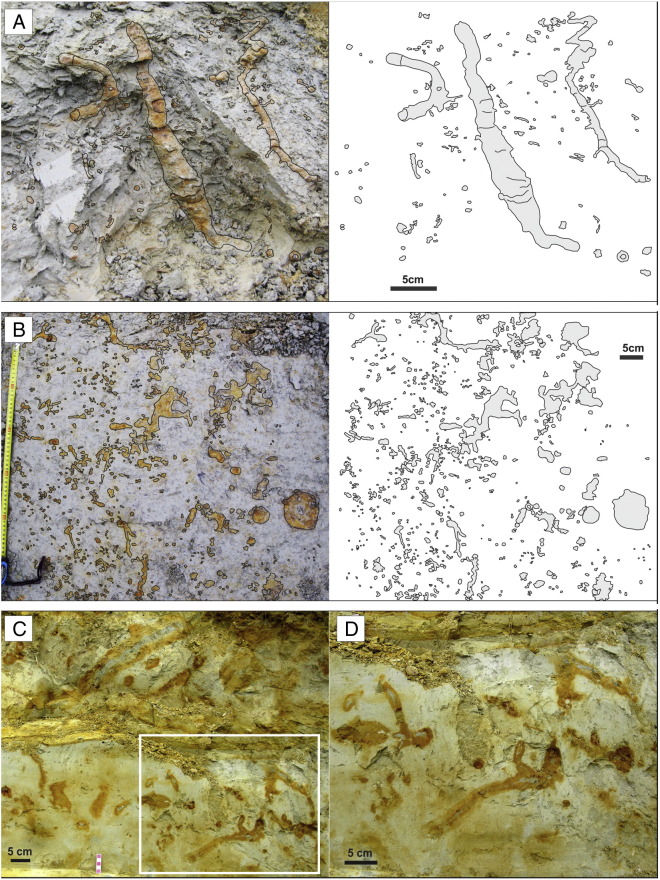
Trace fossils and bioturbation at the Gbely section. (A) Lower bioturbated interval (BI 1), vertical section. (B) Lower bioturbated interval (BI 1), horizontal section; note horizontal branching. (C) Upper bioturbated interval (BI 2), vertical section, with a closer view of a selected part (D).

The lower bioturbated interval (BI 1 = FA 6 + FA 7) represented by brown-grey clayey and silty beds has yielded completely and partially lithified trace fossils (Figs. 3A–B). These beds reflect quiet deposition from suspension in lacustrine embayments (FA 6) and coarser sedimentary input (silt to fine sand) that may represent storm deposits or turbid underflows discharged directly from fluvial or deltaic distributaries during large floods (FA 7). BI 1 corresponds to the *Congeria subglobosa* Beds as referred to in Starek et al. (2010).

**Fig. 4:**
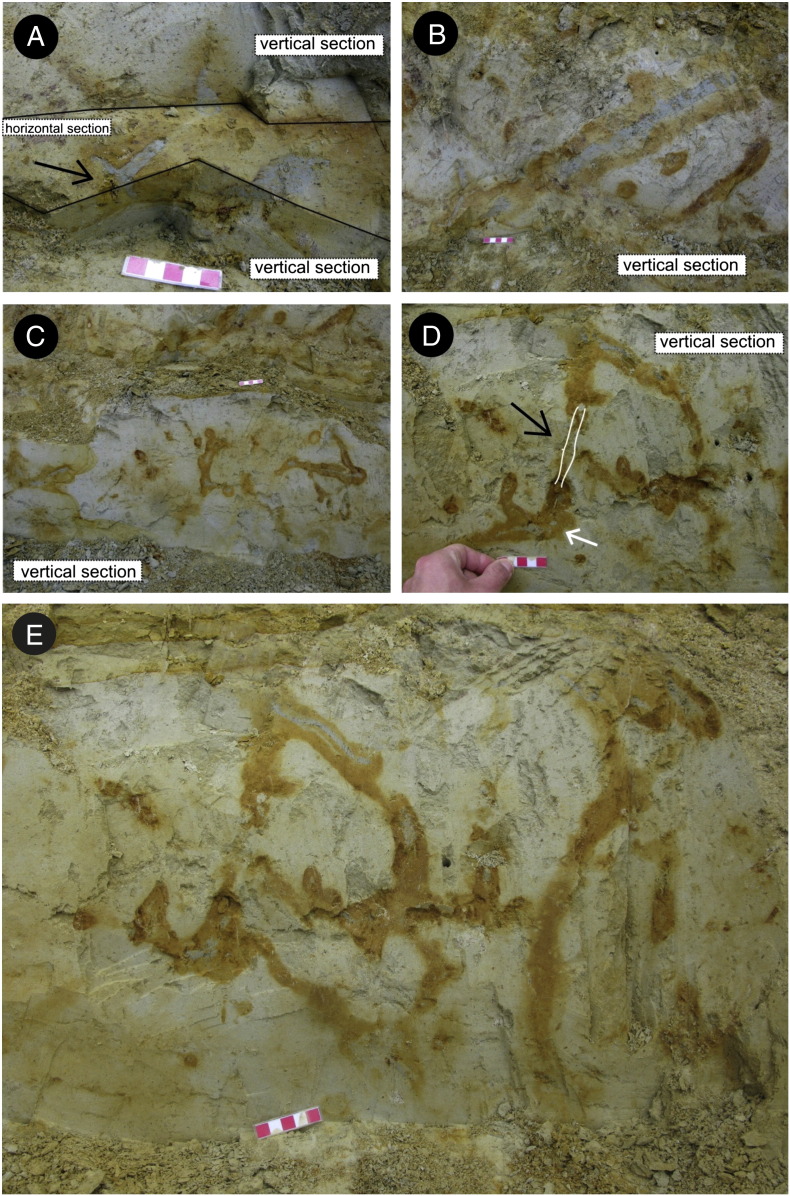
Trace fossils and bioturbation at the Gbely section. (A) Burrow branched horizontally (black arrow). (B–C) Burrow branched vertically upward. (D) Upward and downward branching. Black arrow points to supposed connection between two Y-shaped structures. White arrow indicates branching in horizontal direction. (E) Different view on the same freshly excavated section wall as in D.

The upper bioturbated interval (BI 2 = FA 8B + FA 8C) represented by rhythmic deposition of silty clay, silt, and fine sand reflecting successive progradation of a prodelta (FA 8B) and laterally migrating distal bars (FA 8C) contains unlithified trace fossils (Figs. 3C–D, 4).

### 2.3 Palaeoenvironment

The brackish-water sedimentary environment in Lake Pannon is documented by stable isotopes (Geary et al., 1989; Harzhauser et al., 2007) and ostracod and mollusc associations (e.g. Kováč et al., 1998; Pipík, 1998; Cziczer et al., 2009; Starek et al., 2010). The salinity of offshore waters ranged from 10–15 ‰. Only marginal parts of the lake that were influenced by rivers and by deposition during the terminal stage of Lake Pannon are represented by sediments with a fauna that documents salinities of 0–15 ‰ or freshwater environments (Kováč et al., 1998, 2005). The Gbely sequence was deposited in a brackish-water environment influenced by freshwater input. The salinity can be determined as 3–15‰ on the basis of the presence of the ostracod genera *Cyprideis, Euxinocythere*, and *Loxoconcha* (Starek et al., 2010). The analysed sediments consist of clay, silt, and very fine sand. They are interpreted as part of a brackish lacustrine-deltaic sequence deposited below the fair-weather wave-base (Starek et al., 2010).

### 2.4 Material and methods

Lithified trace fossils were taken from the outcrop with documented orientations. Subsequently, trace fossils were packed in plastic bags to prevent desiccation. Nevertheless, considerable material was destroyed: poorly lithified casts disintegrated.

A surface of each preserved specimen (burrow fragment) was cleaned of clay by slow running water and a paintbrush. Wet casts were impregnated immediately by an acrylic sealer. The acrylic sealer hardened trace fossils and prevented further damage and cracking. Unlithified trace fossils were documented by taking photographs of vertical and horizontal serial sections in outcrops.

Isolated burrow parts were oriented in the position they were found in the section and compared with extant and fossil burrow systems. This procedure provided clues to interpretation of the studied material (see chapter 4.1.1).

The ichnoassemblage of the studied section seemingly consists of several different structures (Fig. 4). After close examination, however, most of them were identified as parts of the single trace fossil (compound structure *sensu* Bertling et al., 2006). We refrain to keep this prominent trace fossil in open nomenclature as it possesses distinct features and so far it has not been reported from the deposits of the Pannon Lake. Thus, a new genus and species are proposed for this trace fossil.

## 3 Systematic ichnology

The material examined herein is deposited at the Natural History Museum of the Slovak National Museum in Bratislava (Slovakia) under the repository numbers SNM-Z 24165, SNM-Z 37735–37747.

### 3.1 *Egbellichnus* igen. nov.

Etymology: after *Egbell*, the Hungarian and German name for the town Gbely (close to the type locality) and ιχνος, from the Greek, meaning trace.

Diagnosis: Vertical, inclined, or horizontal cylindrical burrows of constant diameter without wall structure. The burrows are circular in cross-section. An inclined component typically slopes at ca. 45°; tunnels make loops resembling a spiral or bend at approximately right angles; the resulting burrow shape resembles a corkscrew or an irregular zigzag pattern depending on alternating directions. First to second and more orders of upward, downward or horizontal Y-shaped branching may occur on main burrows. Points of bifurcation and bending have usually an enlarged diameter.

Type ichnospecies: *Egbellichnus jordidegiberti* isp. nov. (only known ichnospecies).

Remarks: This ichnogenus is distinguished from other related ichnotaxa by a combination of typically inclined (roughly at 45°) cylindrical burrows, absence of lining, and tunnels making loops or bends.

For *Thalassinoides* Y- or T-branching is typical, thus, resembling *Egbellichnus*, however, a complex pattern of loops and bendings exhibitied by the latter ichnogenus is absent in *Thalassinoides*.

For *Gyrolithes*, a tunnel forms a dextral or sinistral circular helix with rather constant radius of whorls (Bromley and Frey, 1974; Fillion and Pickerill, 1990), whereas in *Egbellichnus* the loops are irregular and bendings often are in the right angle.

*Egbellichnus* clearly represents parts of a larger three-dimensional open burrow system which was subsequently passively filled with sediment. As such, the resulted trace fossil is considered a compound structure. Similarly inclined burrow tunnels can be produced by several crab taxa (e.g. Gecarcinidae, Macrophthalmidae, Ocypodidae); these, however, usually lack branchings (Vannini, 1980).

Simple fragments or vertical shafts of *Egbellichnus* are slightly reminiscent to the *Dreissenomya* burrows described from Lake Pannon (e.g. Magyar et al., 2006). Presence of inclined burrow parts, branchings and spirally-shaped components in *Egbellichnus* and their absence in mollusc burrows clearly distinguish both ichnofossils from each other.

### 3.2 *Egbellichnus jordidegiberti* isp. nov.

2010. “Thalassinidean (types 1 and 2) burrows“. Starek, Pipík, and Hagarová. p. 379.

**Fig. 5:**
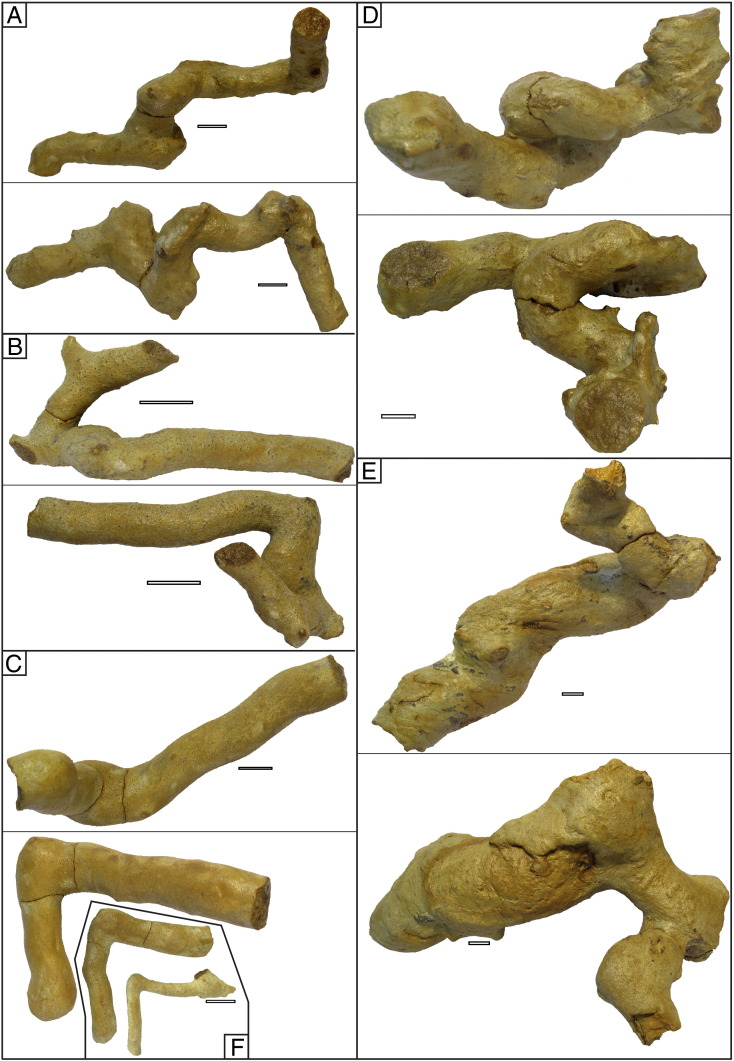
*Egbellichnus jordidegiberti* igen et isp. nov. (A) Holotype SNM-Z 37741. (B) Paratype SNM-Z 37742. (C) Paratype SNM-Z 37743. (D) Paratype SNM-Z 37744. (E) Paratype SNM-Z 37745. (F) SNM-Z 37739 (top), SNM-Z 37740 (bottom). Top parts of the figures depict tunnels oriented in side view (*in situ* position), below they are viewed from above. Scale bar equals 10 mm.

**Fig. 6:**
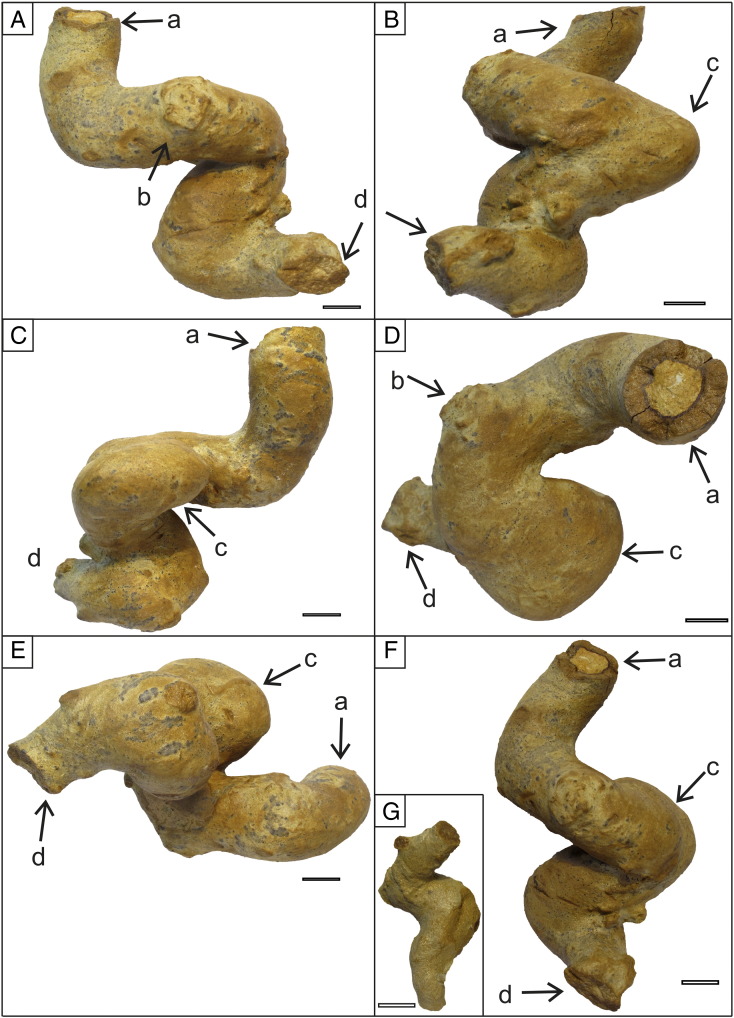
*Egbellichnus jordidegiberti* igen et isp. nov., paratype SNM-Z 37738 viewed from different angles. (A–C) Side views oriented in the *in situ* position. Scale bar equals 10 mm.

**Fig. 7:**
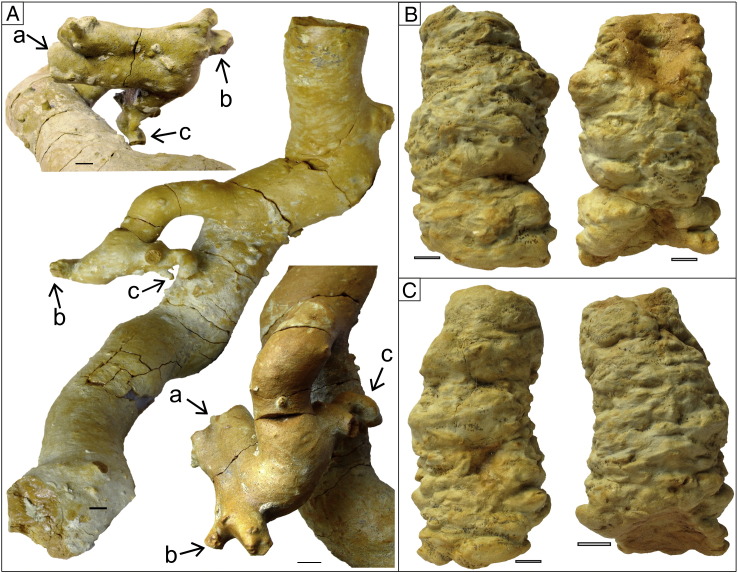
Ichnoassemblage at the Gbely section. (A) *Egbellichnus jordidegiberti* igen et isp. nov., large burrow part exhibiting lateral tunnel branching downward (SNM-Z 24165); the figure in the middle is oriented in *in situ* position. (B) Vertical cylindrical shaft with knobby surface, SNM-Z 37746. (C) Vertical cylindrical shaft with knobby surface, SNM-Z 37747. Scale bar equals 10 mm.

Etymology: After Jordi M. de Gibert, an enthusiastic ichnologist, a friend and collegue of the authors, who suddently passed away in September 2012.
Diagnosis: As for the ichnogenus.
Holotype: SNM-Z 37741 (Fig. 5A).
Paratypes: SNM-Z 37738, SNM-Z 37742–37745.
Other material: SNM-Z 24165, SNM-Z 37735–37737, SNM-Z 37739–37740.
Description: The trace fossil consists of unlined, essentially cylindrical components; the diameter may vary between individual components, ranging from 8 to 70 mm (Figs. 5, 6, 7A); a single tunnel usually has a constant diameter for its entire preserved length. The passive fill is homogeneous; tiny, well delineated, concentric ferruginous zones occasionally can be seen on the cross-section (Fig. 6D).

**Fig. 8:**
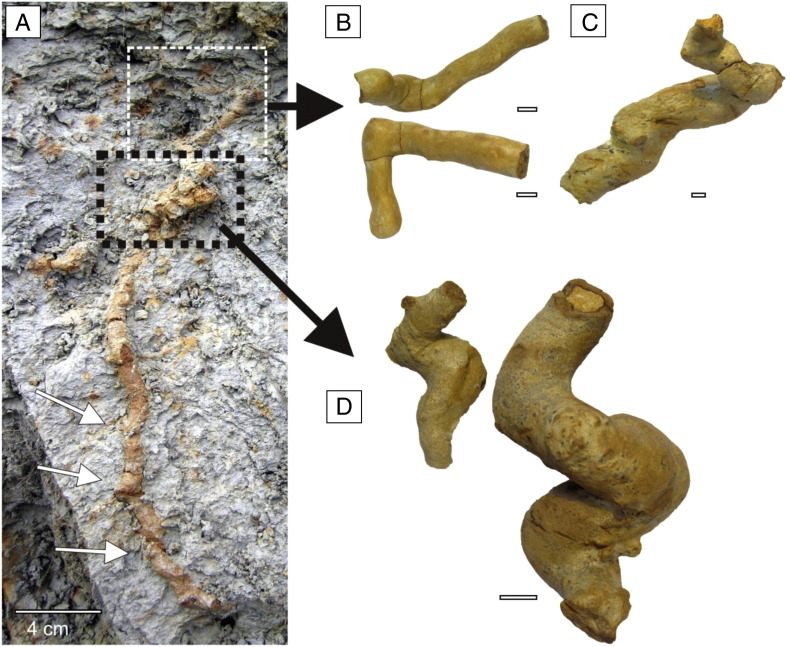
*Egbellichnus jordidegiberti* igen. et isp. nov. (A) Large burrow part *in situ*.White arrows point to enlarged portions at point of bifurcation. In white rectangle an inclined burrow part with right-angle bending is preserved. In black rectangle a spiral-shaped burrow part is preserved. (B–D) Burrow parts analogous to those in A (not the same specimens). Scale bar equals 10 mm.

**Fig. 9:**
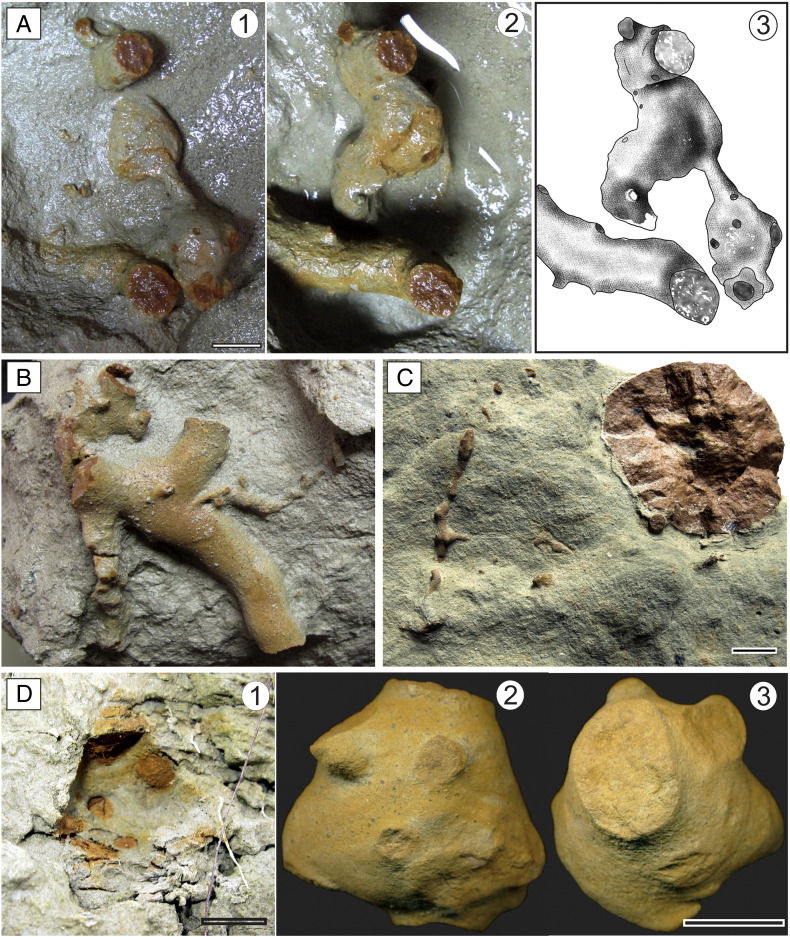
*Egbellichnus jordidegiberti* igen. et isp. nov. (A) Burrow part containing a spherical chamber with several tiny shafts (SNM-Z 37735). The chamber was connected to a burrow system (A1); it disintegrated during the preparation process (A2); A3: Line drawing of the same structure. (B) Horizontal branching (SNM-Z 37736); note tiny tunnels attached to a larger burrow. (C) Cross-section of SNM-Z 24165 (see Fig. 7A). (D) Chamber cast with thin shafts (SNM-Z 37737), D1: *in situ* position in the outcrop, D2: side view, D3: view from above. Scale bar equals 10 mm.

The trace is composed of several parts (Fig. 8). The horizontal component often shows branching; two forms have been observed. The first type can be considered a true *Thalassinoides*-like branching (Figs. 5B, 9B). The second type seems to be a successive branching, i.e. it consists of two tunnels of different diameter (Fig. 5E) although interpreting these structures as preservational artefacts cannot be excluded. Regular rounded winding (meandering) of individual simple tunnels in a horizontal direction without branching has not been observed. The subvertical component consists of simple shafts connecting parts of the burrow system. The inclined component is the most distinctive feature of this ichnotaxon. This component consists of tunnels or shafts which are inclined typically at an angle of about 45° to the horizontal surface. Two main morphologies can be recognized in this component; the tunnel makes loops resembling spirally shaped *Gyrolithes*-like burrows, or/and the tunnel turns at right angle (Figs. 5C, F). If the first component prevails the burrow shape resembles a corkscrew (Fig. 6); if the second prevails, the shape follows an irregular zigzag pattern (Fig. 5B). The two morphologies can alternate.

Commonly at points of bifurcation the diameter of a burrow is enlarged (Fig. 8A). Such enlargement is also present in inclined tunnels that bend horizontally at 90° (Figs. 5C, F).

Spherical chambers with radiating shafts are directly connected to the burrow system (Figs. 9A, D). The diameter of radiating shafts ranges from 1.5 mm to 8 mm, whereas the diameter of chambers ranges from 15 mm to 22 mm.

Remarks: *Egbellichnus jordidegiberti* isp. nov. is considered a typical compound trace fossil which is a result from the changing behaviour of a single producer. Bertling et al. (2006) noted it can represent two different situations: successive or simultaneous formation. In the case of *E. jordidegiberti* isp. nov. we are dealing with a burrow structure formed simultaneously as documented by uniform infill of the trace fossils.

The burrow diameter is virtually always constant within the same tunnel suggesting that each burrow was inhabited by one (or only a few) animal(s). In some cases the diameter of side branches is noticeably smaller (8–20 mm) than the main burrow (Fig. 5E).

One burrow is evidently branched downward (Fig. 7A). The burrow length was estimated according to field observation to have been about 1.5 m to more than 2 m. Collected fragments of one specimen attained a length of 52 cm (Fig. 7A).

Spherical chambers with radiating shafts can be compared to *Maiakarichnus currani* Verde and Martínez, 2004. Unlike *Maiakarichnus* the chamber is more regularly spherical and shafts radiate in all directions upward and downward; in *Maiakarichnus* thin shafts radiate mainly in stratigraphically upward direction from the upper part and from sides (Verde and Martínez, 2004). Possibility that the chambers represent concretions can be excluded. Concretions are rounded masses of mineral matter found in sedimentary rock; the chambers described herein have the same sedimentary filling as other lithified trace fossils and are connected to the main burrow (Fig. 9A). Based on the criteria given by Pickerill (1994), the chambers are considered an ethological structure: 1) they are of uniform size; 2) they occur as a regular, complex and repetitive geometric form; 3) they possess very delicate morphologic features; 4) and they are preserved in full relief. The chambers are relatively rare (two collected samples and four field observations; Fig. 9) but they were found in both bioturbated intervals of the studied section.

In addition to the true branching points passive “junctions” of burrow branches also were observed; these, however, are considered to be the result of taphonomic processes. Preserved burrow casts have a greater diameter than the tunnels themselves; thus, when two tunnels (or parts of a single tunnel) were close to each other, the burrow walls nearly touch each other.

No trace fossil reported from the Lake Pannon previously (Jámbor, 1980, 1987; Babinszski et al., 2003; Cziczer et al., 2009) can be directly compared to *E. jordidegiberti* isp. nov.

Besides *Egbellichnus jordidegiberti* n. igen. n. isp., several other components have been recognized in the studied section. In BI 1, vertical cylindrical shafts with knobby surface and a diameter of around 3–5 cm rarely occur. Knobs are oriented horizontally and they clearly represent bioglyphs (Figs. 7B–C). This trace fossil probably is part of a larger and more complex burrow system of *Egbellichnus*; but, because it was found only in BI 1 the trace fossil characteristics are not included in the description of *Egbellichnus*. Moreover, the preservation of bioglyphs is atypical for the entire ichnoassemblage.

In close proximity to the *Egbellichnus* burrows, small shafts with diameters of 1–3 mm occur. They form a maze of tiny tubes; some of them are connected (perpendicularly or obliquely) to larger burrows (Fig. 7A).

In the uppermost part of BI 2 there is a continuous transition to the succession with preserved equilibrichnia with mechanical collapse and fluid-upwelling structures (Starek et al., 2010). It is possible that formation of some of these mechanical structures were triggered by animal escape activity. Vertical equilibrichnia possibly represent escape structures of bivalves, most probably *Dreissenomya*, although no shells were found (cf. Starek et al., 2010).

In the bioturbated intervals, no vertical partitioning of the community was observed. Both, in the vertical and horizontal aspect, the trace-fossil assemblage is uniform.

### 3.3 Mode of preservation.

The trace fossils are preserved either as lithified yellowish casts of burrows (without preserved bioglyphs) in dark clayey sediment or as unlithified trace fossils in silty/sandy sediments. Associations of both preservation types are composed of very similar trace fossil assemblage; i.e. both bioturbated horizons are dominated by the newly recognized trace fossil *Egbellichnus jordidegiberti* n. igen. n. isp. In the surrounding dark clay of BI 1 partially or completely lithified yellowish trace fossils are clearly visible (Fig. 3A). The fill is composed of silt with a better potential for lithification than the surrounding plastic clay and silty clay. In BI 2 coarser siltstone to sandstone beds with unlithified trace fossils are exposed (Figs. 3C–D). Trace fossils are readily distinguished from the surrounding sediment due to the halo effect of burrows (Bromley, 1996). The largest burrows also contain a dark clay filling. Walls or lining were not recognized.

Lithification of trace fossils was probably caused by a fluid migration inside the burrow systems. Predisposition for easier fluid migration was supported by open burrow systems and coarse filling of burrows. Lithified silty/sandy burrow casts were cemented by ferruginous and calcareous compounds. Ferruginous concentric structures are visible on transverse cuts of burrows (Figs. 3B, 6D, F). Cross sections of the horizontal and subhorizontal burrows are elliptic to circular, thus, compaction had no significant effect on the morphology of the trace fossils. Sections with lithified trace fossils contain also sporadically lithified thin beds. Similar ferruginous preservation of trace fossils was reported from Miocene sandy silts of southern Spain (Muñiz et al., 2010; de Gibert et al., 2013).

The light yellow/brown colour of the burrow walls produced in the Gbely section by early diagenetic pigmenation from pore water indicates more oxidizing conditions in the burrow than in the subsurface sediments. Such conditions are typical for ghost shrimp burrows, in which physico-chemical and microbial properties are more similar to the surface sediments than to the surrounding subsurface sediments (Bird et al., 2000). In this respect the mode of preservation is in agreement with the tracemaker identification as discussed in chapter 4.1.

## 4 Discussion

### 4.1 Identification of tracemaker

The trace fossils described above show a suite of characters which is typically interpreted as arthropod burrows generally attributed to crustaceans (e.g. Frey et al., 1984; Bromley, 1996; Bishop and Williams, 2005; Seilacher, 2007). One of these characters, Y- or T-shaped branching, can be occasionally attributed to invertebrate groups different from crustaceans (e.g. some enteropneusts, echiurans; see Pervesler and Uchman, 2009: p. 141). However, if present, such Y-shaped branching is usually not part of a larger complex burrow system and is positioned vertically. In the studied section, the trace fossils (especially unlithified) may superficially look like simple upright Y-shaped structures (Figs. 3C–D, 4D–E), and thus, resembling *Parmaichnus*, *Polykladichnus* or *Psilonichnus*, especially on freshly excavated section wall. Removing the sediment, however, clearly showed that in our case the shafts and tunnels are parts of larger burrow system, and thus, they represent a compound structure. The tunnels are connected to each other and simultaneously they attain roughly the same diameter. In conclusion, the morphology of studied burrows strongly suggests that the tracemaker is a crustacean, possibly a malacostracan.

Among modern malacostracans there are basically two higher taxa, Decapoda and Stomatopoda, capable of producing large burrows comparable with those described herein. Stomatopods construct shallow and rather simple burrows (Myers, 1979). Thus, it seems that decapod crustaceans are the most likely tracemakers.

There are several higher taxa of decapod crustaceans in which the construction of permanent burrows or burrow systems evolved independently. Unfortunately, identifying decapods as producers of burrows without direct evidence of *in situ* preservation is rather difficult; ichnofossils commonly attributed to decapod crustaceans usually do not contain any body fossils. Such associations are rare (Stilwell et al., 1997; Bishop and Williams, 2005; Hyžný, 2011; Hyžný and Hudáčková, 2012). Species that produce permanent burrows have been identified in six decapod infraorders *sensu* De Grave et al. (2009): Caridea (the family Alpheidae only), Astacidea, Glypheidea, Gebiidea, Axiidea, and Brachyura (e.g. families Gecarcinucidae, Goneplacidae, Portunidae, Panopeidae, Gecarcinidae, Sesarmidae, Varunidae, Dotiliidae, Macrophthalmidae, Mictyridae, and Ocypodidae). The most complex burrow systems are constructed by members of the infraorders Gebiidea and Axiidea (in the past collectively known as thalassinideans, see Dworschak et al., 2012; Poore et al., 2014). Extant members of Laomediidae and Upogebiidae (Gebiidea), and of Axianassidae and Callianassidae (Axiidea) are known to construct very complex burrow systems, some of which can reach more than 1 metre in depth (e.g. Dworschak and Ott, 1993).

Geometrically complex burrow systems comparable to the material presented herein are produced only by the Alpheidae (e.g. Shinn, 1968: pl. 109, Fig. 1; Dworschak and Pervesler, 2002: Fig. 1), some crayfishes (Astacidea) (Hasiotis and Mitchell, 1993), and axiideans and gebiideans (Griffis and Suchanek, 1991; Dworschak and Ott, 1993; Nickell and Atkinson, 1995; Dworschak, 2004). These three decapod groups also have been identified as excavating their burrows in a similar manner (Atkinson and Taylor, 1988). We argue that in the present case the presumed burrow morphologies of studied samples can narrow significantly the identity of the tracemaker. In this respect, we use basically two independent lines of evidence: 1) interpreting the tracemaker based on the burrow morphology itself and 2) inferring the tracemaker from the ecological conditions interpreted from the study of other animal groups and sedimentology.

#### 4.1.1 Burrow morphology

The presence of Y or T branching (positioned either horizontally or vertically), spirally shaped tunnels when viewed from above, more-or-less constant diameter of most burrow-system tunnels with swellings interpreted in some taxa as turning chambers, are all characters typical for axiidean and gebiidean shrimps. Members of other decapod groups usually produce simpler burrows with limited branching (e.g. marine Astacidea, supratidal and intertidal brachyuran crabs) or rather complex horizontal mazes (e.g. the caridean family Alpheidae, or the brachyuran crab *Goneplax rhomboides*). In contrast, highly structured burrow systems are typical for axiidean and gebiidean shrimps. They are considered to be producers of the most complex burrow systems in the entire animal kingdom, and they construct species-specific burrows (Griffis and Suchanek, 1991; Dworschak and Ott, 1993; Nickell and Atkinson, 1995; Felder, 2001; Dworschak, 2004). Griffis and Suchanek (1991) proposed a simple model to classify their architecture and trophic mode. Nickell and Atkinson (1995) criticised the model for being too simplistic. De Gibert and Ekdale (2010) pointed out difficulties with applying the burrow classification of Griffis and Suchanek (1991) in the trace fossil record, because it is usually hard to isolate individual burrow systems and mounds at the openings and number of openings are usually not preserved.

Trace fossils roughly similar to *Egbellichnus* from freshwater environments and interpreted as crayfish burrows have been assigned to different ichnogenera (Hasiotis and Mitchell, 1993; Zonneveld et al., 2006; Bedatou et al., 2008; see Bedatou et al., 2008 for their review). Crayfish burrow morphologies include both simple and complex architectures with varying degrees of branching, chamber and vertical shafts development (Hobbs, 1981; Hasiotis and Mitchell, 1993). They typically exhibit surficial morphologies, i.e. bioglyphs (Hasiotis and Mitchell, 1993), which are missing in our material. The preservation of bioglyphs is related to the consistency of the substrate (Seike and Nara, 2007); thus, their absence in our material can be explained in the terms of the nature of the sediment. However, crayfish burrows are characteristic of continental rather than marine-influenced facies (Hasiotis and Mitchell, 1993) and are never built in the subtidal zone (Hobbs, 1981).

The studied vertical and subvertical burrow system parts are reminiscent of burrows of brachyuran crabs exemplified by members of the families Gecarcinidae, Ocypodidae, and Sesarmidae (Vannini, 1980; Seike and Nara, 2008). Branching is, however, uncommon in burrows of these taxa and complex burrows comparable to our material were reported only in a few species (Vannini, 1980). It is important to note that crab burrows with morphology comparable to the studied material are known from intertidal environment, whereas *Egbellichnus* clearly originated in the sublittoral zone (Starek et al., 2010).

Of the gebiideans, only burrows of the Axianassidae (*Axianassa*), Laomediidae (*Jaxea*), and Upogebiidae (*Upogebia*) are sufficiently known. Burrows of *Axianassa australis* are characterized by spiral vertical shafts (*Gyrolithes*-like) leading to wide horizontal galleries from which several evenly proportioned corkscrew-shaped spirals branch off and lead to further horizontal galleries at greater sediment depths (Dworschak and Rodrigues, 1997; see also Felder, 2001). Spirally shaped tunnels in our material are not positioned vertically as in *Axianassa* burrows, but rather subvertically with the axis of the corkscrew shape inclined at about 45 degrees. Moreover, no widened galleries have been identified in our material.

The laomediid *Jaxea* produces burrow systems with rather large swollen chambers – galleries connected to horizontal and subhorizontal tunnels of smaller diameter (Pervesler and Dworschak, 1985). However, the diameter itself does not correspond to the body of an animal and changes throughout the entire burrow system. Presence of large galleries and changing burrow diameter is not consistent with *Egbellichnus*.

The Upogebiidae generally produce vertical Y-shaped burrows consisting of a U- or double U-shaped gallery and a basal vertical shaft (e.g. Ott et al., 1976; Dworschak, 1983; Curran and Martin, 2003). Recently, Pervesler and Uchman (2009) identified upogebiid burrows in the Pleistocene of Italy. They discussed identifying Upogebiidae as a tracemaker in the fossil record and stated that „fossil burrows without turning chambers should not be attributed to the work of upogebiids“ (Pervesler and Uchman, 2009: p. 139). As discussed in their paper, turning chambers in the upper part of the burrow are an obligatory feature of *Upogebia* burrows. Addressing this issue, it is important to note that identifying turning chambers in the fossil record depends on the interpreted functional morphology of the studied burrow. Thus, the term itself mirrors the function of a chamber, not its general morphology. Therefore, turning chambers can be difficult to identify in the fossil burrow systems. What really is typical for upogebiid burrows is the presence of turning chambers in the upper part of a burrow (Griffis and Suchanek, 1991: Fig. 1; Pervesler and Uchman, 2009: Figs. 5I–K); that is, in the shaft before the junction with a basal shaft (see above). No such structure can be identified in *Egbellichnus*.

Among axiideans, the Axiidae (*Axius*, *Axiopsis*) and Strahlaxiidae (*Neaxius*) seem to construct rather simple burrows without complex morphology (Pemberton et al., 1976; Dworschak and Ott, 1993; Kneer et al., 2008; Vonk et al., 2008). In contrast, members of the Callianassidae construct the most complex and extensive burrow systems of all known fossorial shrimps. Thus, with respect to the discussion above, it is probable that the producer of the studied burrows is a member of the Callianassidae. Although closer assignment is not currently possible, we compared the architecture of callianassid burrows with our material and found some striking similarities.

**Fig. 10:**
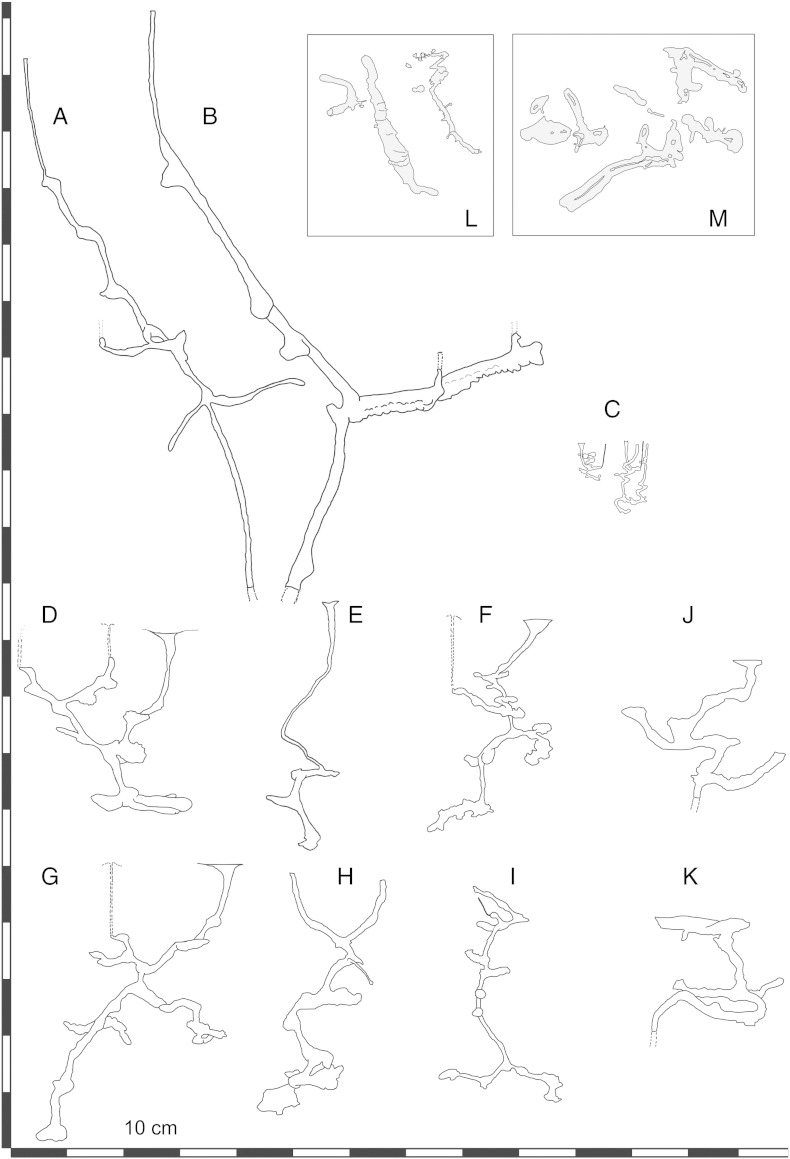
Burrow morphology of extant callianassid ghost shrimps compared to *Egbellichnus jordidegiberti* igen. et isp. nov. (A–B) *Lepidophthalmus louisianensis*, after Dworschak (2000b). (C) *Paratrypaea bouvieri*, after Dworschak and Pervesler (1988). (D–I) *Pestarella tyrrhena*; D, G, F after Dworschak et al. (2006); E after Dworschak (1987); H, I after Dworschak (2001). (J–K) *Pestarella whitei*, after Dworschak (2002). (L–M) *Egbellichnus jordidegiberti* igen. et isp. nov. as preserved in the studied section, see Fig. 3A. All burrow schemes are to scale.

Burrow parts shaped similarly to that of the *Egbellichnus* burrow fragments have been identified in a handful of callianassid genera classified within the Callianassinae: *Biffarius*, *Callianassa*, *Paratrypaea*, and *Pestarella*; and one genus of the Callichirinae: *Lepidophthalmus*. Burrows of these callianassine genera possess inclined, spirally shaped main tunnels often with angular turnings with slightly swollen areas (which may be interpreted as turning chambers). The burrow itself is more complex in vertical aspect with branching occurring mainly in horizontal aspect. Such morphologies (Fig. 10) have been fully, or at least partly, described in *Biffarius filholi* (Berkenbusch and Rowden, 2000: Fig. 2), *Callianassa truncata* (Ziebis et al., 1996: Fig. 2), *Paratrypaea bouvieri* (Dworschak and Pervesler, 1988: pl. 2), and several species of *Pestarella*: *P. candida* (Dworschak, 2002: Fig. 2a, b), *P. tyrrhena* (Dworschak, 1987: Fig. 1b; 2001: Fig. 2; Dworschak et al., 2006: Fig. 1; Koller et al., 2006: Fig. 1) and *P. whitei* (Dworschak, 2002: Fig. 2c, d). Finally, Dworschak (2000b: Fig. 3) described burrows of *Lepidophthalmus louisianensis* which are similar to *Egbellichnus* burrows in posssessing rather long subvertical main shafts with ramified horizontal side branches.

Thus, *Egbellichnus* is interpreted as a decapod dwelling trace. In addition, the studied burrow systems show rather low morphological diversity suggesting the producers to be members of a single species, most probably a callianassid ghost shrimp.

#### 4.1.2 Tolerance of brackish environments.

Ichnoassemblages of brackish-water environments typically are taxonomically impoverished in comparison with fully marine deposits. They are characterized by several features, notably by low ichnodiversity, dominance of infaunal traces rather than epifaunal trails, simple structures produced by trophic generalists, and presence of monospecific suites (Wightman et al., 1987; Pemberton and Wightman, 1992). Brackish-water ichnofaunas reported from the Pliocene strata of Spain and France (e.g. Muñiz and Mayoral, 1998) are commonly depauperate and dominated by crustacean burrows. The same is true for the Gbely section. Buatois et al. (2005) noted that Miocene estuarine deposits exhibit smaller trace fossils attributed to annelids, but not necessarily smaller burrows constructed by crustaceans.

There is only a limited number of invertebrate animals which are able to construct complex burrow system in the brackish environment; thus, in an environment identified at the studied locality. It is obvious that the tracemaker must have been an animal able to tolerate brackish waters with fluctuating salinity. From macrocrustaceans constructing complex burrows only few groups are able to tolerate reduced salinity for long periods.

Alpheid shrimps usually inhabit marine, shallow tropical and subtropical waters (Chace, 1988), only a few have colonized oligohaline or freshwater habitats (Yeo and Ng, 1996). Their burrow diameter is relatively small (e.g. Dworschak and Pervesler, 2002) in comparison with *Egbellichnus* from Gbely. In this respect it is worth noting that large interconnected burrows from the salt marshes of Georgia originally interpreted as being produced by alpheid shrimps (Basan and Frey, 1977) were re-interpreted as being made by a mud crab, *Panopeus herbstii* (Martin, 2013).

Virtually all crayfish are freshwater animals tolerating low salinity fluctuations (Crandall and Buhay, 2008) and many burrow in soil with a connection to the water table (Hobbs, 1981; Hasiotis and Mitchell, 1993). Although *Caspiastacus* from the Caspian Sea lives in brackish water (12–13‰), it is not known to make complex permanent burrows, and it reaches its greatest abundance at a depth of ca. 10 m (Cherkashina, 1999).

There are several brachyuran families inhabiting brackish-water environments. Of burrowing taxa, the family Ocypodidae is the most familiar. Ocypodids, however, do not construct complex burrow systems and are partly connected to the marine environment, where they release their eggs. Moreover the burrow morphology itself (Vannini, 1980; de Gibert et al., 2013) does not fully correspond to *Egbellichnus*. Burrowing crabs are typical of intertidal areas (upper-intertidal zone), and they do not build their burrows below the fair-weather wave-base (e.g. Vannini, 1980; Netto and Grangeiro, 2009; de Gibert et al., 2013), as is the case in *Egbellichnus*.

Axiideans and gebiideans are known to tolerate relatively high salinity fluctuations also inhabiting brackish-water environments (see below). Thus, we argue for an axiidean or gebiidean decapod crustacean as the tracemaker. Unfortunately, no detailed analysis of the distribution of axiidean and gebiidean species in relation to salinity exists, as this parameter is usually not recorded or mentioned in the literature. There are some species which prefer marine habitats, whereas others are found in brackish waters (Dworschak, 2000a, 2005).

Many axiidean and gebiidean species are known to inhabit estuaries; thus, they live in an environment both under fluvial (freshwater) and tidal influences. For instance, the Upogebiidae are able to tolerate a salinity range from fully marine (36 ‰) to salinity of 9 ‰ in freshwater-influenced areas (Dworschak, 1987). In this respect the callianassid genus *Lepidophthalmus* is the most extreme example as it is able to tolerate freshwater environments (Dworschak, 2007). Generally it is concentrated in intertidal and shallow subtidal substrates ranging from sandy mud to organic silty sand. Felder and Lovett (1989) characterized *Lepidophthalmus louisianensis* as adapted to oligohaline habitats of coastal marshes, tidal channels and estuarine embayments. In this respect, osmoregulatory adaptations of adults and larvae were studied by Felder (1978) and Felder et al. (1986). They are also known to tolerate periodic anoxia in their burrows (Felder, 1979). *Callianassa kraussi* (some authors classify it as a member of *Callichirus*, see Hyžný and Müller, 2010) is considered to be the ecological equivalent of *L. louisianensis* in South Africa (Felder and Lovett, 1989). *Lepidophthalmus turneranus* has been reported to migrate up rivers in West Africa (Vanhöffen, 1911).

Thus, it seems most reasonable to assume that the tracemaker of *Egbellichnus jordidegiberti* was an axiidean or gebiidean shrimp, most probably a member of the family Callianassidae.

### 4.2 Biological aspects of the tracemaker

Among callianassid ghost shrimps, several life strategies have been adopted (Griffis and Suchanek, 1991; Felder, 2001), but because of the fragmentary nature of the *Egbellichnus* burrow system we refrain from inferring the main trophic mode of the tracemaker. Nevertheless, some phenomena commonly related with callianassid life strategies can be mentioned and briefly discussed.

#### 4.2.1 Juvenile-adult association of tracemakers

The *Egbellichnus* association exhibits burrows with variable diameter but the same morphological features. We interpret them here as a juvenile-adult association. Association of juvenile and adult burrows has been reported previously in *Upogebia affinis* (Frey and Howard, 1975) and *Nihonotrypaea japonica* (Tamaki and Ingole, 1993). Several examples of juvenile-adult association are known in the fossil record of axiidean (or other crustacean) burrows; Howard (1966) described such an association for *Thalassinoides* from the Cretaceous of Utah; Curran (1985) for *Ophiomorpha* from the Cretaceous of Delaware; de Gibert (1996) and de Gibert et al. (1999) described the same for *Sinusichnus* from the Pliocene of Spain and France. From extant environments, bimodality in populations has been documented in *N. japonica* (Tamaki and Ingole, 1993) and *Biffarius filholi* (Berkenbusch and Rowden, 1998). More discussion on this topic with respect to the fossil record was provided by de Gibert et al. (2006).

A juvenile-adult association is occasionally connected with utilization of adult burrows by juveniles. In callianassids this behaviour has been reported in several extant taxa: *Callianassa kraussi* (Forbes, 1973), *Neotrypaea californiensis* (Swinbanks, 1981), and *N. japonica* (Tamaki et al., 1992; Tamaki and Ingole, 1993). Usually it can be identified in burrow casts as juvenile branches lead off from a stouter, adult structure. de Gibert et al. (2006) reported this phenomenon in the fossil record (*Ophiomorpha puerilis* from the Pleistocene of Brazil). The tunnels with distinctly smaller diameter attached to larger *Egbellichnus* burrow system parts (Fig. 7A) may actually represent the juvenile occupancy.

Forbes (1973) and Frey and Howard (1975) described juvenile burrows as originating in adult burrow chambers that they interpreted to be brood chambers. Similarly, in the fossil record, Curran (1976) and Curran and Frey (1977) described *Ophiomorpha* from the Pleistocene of North Carolina as possible callianassid brood structures. Later, Verde and Martínez (2004) described the same structures from the Miocene of Uruguay and on its basis formally erected the new ichnogenus *Maiakarichnus*. Spherical chambers with radiating shafts of *Egbellichnus* (Fig. 9) are reminiscent of *Maiakarichnus* but differ in having shafts radiating in all directions. Because only a limited number of samples with such morphology has been recovered, we are reluctant to further speculate on their function, and therefore, we accept interpretation of them as brood structures of the supposed callianassid tracemaker.

#### 4.2.2 Commensalism

A maze of tiny tubes is occassionally preserved in the surroundings of the *Egbellichnus* burrow system. These tubes may be attached directly to the tunnels themselves and if considered them as contemporaneous the smaller tubes can be interpreted as having been produced by commensal organisms, such as worms, living in direct proximity to or within decapod crustacean burrows. Such associations have been observed commonly both in extant habitats (Atkinson and Taylor, 2005) and trace fossil assemblages (e.g. de Gibert et al., 2006). Because large burrows like those of callianassid ghost shrimps influence the surrounding substrate (e.g. Ziebis et al., 1996; Bird et al., 2000) organisms which would normally not occur at the place are present.

Numerous associates and ectosymbionts across different higher taxa are commonly found in burrows of callianassid shimps (and other axiideans and gebiideans) (e.g., Atkinson and Taylor, 2005; Kneer et al., 2008). In salinity-stressed environments, however, limited numbers of commensals can be expected to occur with callianassid burrows. Major infaunal animals that have invaded and tend to dominate brackish systems include bivalves, fossorial shrimps (axiideans and gebiideans), amphipods, polychaetes and threadworms (Buatois et al., 2005). Diminution and lower diversity with higher salinity stress have been documented in modern environments; size reduction, however, typically is displayed by annelid traces, but not necessarily in crustacean burrows (Buatois et al., 2005 and references therein). Thus, polychaetes seem to be the most probable candidates for the producers of tiny tubes associated with *Egbellichnus* burrows.

#### 4.2.3 Survival of ghost shrimps in Lake Pannon

Endemic species are often documented from long-lived brackish (Caspian Sea; e.g. Dumont, 1998) and freshwater lakes (Malili, Tanganyika; e.g. Rintelen and Cai, 2009 and Snoeks, 2000, respectively). During Late Miocene time, the Vienna Basin was an embayment of Lake Pannon which was ecologically comparable with today's Caspian Sea. In fact, a high rate of endemism among several animal groups (Dinoflagellata, Mollusca, Ostracoda) of Lake Pannon has been documented previously (Magyar et al., 1999; Müller et al., 1999; Pipík, 2007; Danielopol et al., 2008; Harzhauser and Mandic, 2008; Cziczer et al., 2009).

Today, no ghost shrimp are known from long-lived brackish lakes. Nevertheless, the fossil record of the Central Paratethys ghost shrimp is rather robust (Hyžný and Müller, 2010, 2012; Hyžný, 2011, 2012a,b; Hyžný and Hudáčková, 2012; Hyžný and Dulai, 2014); thus, their persistence in Lake Pannon after seaway closure can be expected, although no callianassid body fossils from the Upper Miocene of the study area have been found so far. The adaptations of ghost shrimps as discussed in chapter 4.1.2 demonstrate that this animal group has abilities to utilize new ecological niches. Todays salinity-stressed environments are commonly inhabited by ghost shrimps. Also, fossil callianassid remains are known from settings with salinity fluctuations (Turek et al., 1988; Hyžný and Hudáčková, 2012; Hyžný et al., 2012). Turek et al. (1988) even suggested that callianassid ghost shrimps were among the first decapods to colonize brackish environments. In this respect, the morphological similarities between *Egbellichnus* burrow parts and the burrows of extant *Lepidophthalmus* (Fig. 10A–B), invading freshwater habitats are worth noting. *Lepidophthalmus* is known from the Oligocene of the Central Paratethys (Hyžný and Dulai, 2014) and recently the genus also has been documented from Lower Miocene settings (Gašparič and Hyžný, 2014).

Although it is speculative to argue for generic identification of the tracemaker, it can be demonstrated that the ghost shrimp lineage inhabiting present-day environments with great salinity fluctuations also was present in the Central Paratethys. The *Egbellichnus* burrow systems preserved at Gbely, thus, document survival of ghost shrimps long after the closure of all seaways. In this sense, it is the only, although indirect, record of callianassids from brackish lake environments reported thus far.

## 5 Concluding remarks

(1) The Late Miocene ichnoassemblage of the Bzenec Formation exposed at the Gbely section (the Vienna Basin, Slovakia) is characterized by low diversity, relatively simple structures and presence of monospecific suites. These aspects are typical for brackish-water trace fossil assemblages; indeed, the previous study of the section interpreted the settings as a brackish-water environment influenced by freshwater input (Starek et al., 2010). At that time, the Vienna Basin was an embayment of the large, long-lived Lake Pannon.
(2) A new ichnotaxon, *Egbellichnus jordidegiberti* igen. et isp. nov., is described from the Gbely section. *Egbellichnus* is interpreted as part of a complex open burrow system and is distinguished from other related ichnotaxa by a combination of typically inclined cylindrical burrow parts, absence of lining, and tunnels making loops or bends at approximately right angles.
(3) Based on evidence from palaeoenvironmental reconstruction and burrow morphology the tracemaker of *Egbellichnus* is identified as a member of the family Callianassidae. Ghost shrimps of this family are able to tolerate high salinity fluctuations and construct very complex burrow systems.
(4) Today, no ghost shrimps are known from long-lived brackish lakes. *Egbellichnus*, if interpreted correctly, is thus the only, although indirect, evidence for the persistence of a ghost shrimp in a brackish lake.
